# Feasible CT features to distinguish incidental rib enhancement from sclerotic metastasis in patients with malignancies

**DOI:** 10.1007/s00256-024-04609-3

**Published:** 2024-02-16

**Authors:** Qiuxia Yang, Jiahui Xu, Jianyao Zhou, Qiulin Liu, Zhijun Mai, Hui Xie, Xiaohua Ban, Lizhi Liu, Rong Zhang

**Affiliations:** 1 Department of Medical Imaging Center, State Key Laboratory of Oncology in South China, Guangdong Provincial Clinical Research Center for Cancer, Guangdong Key Laboratory of Nasopharyngeal Carcinoma Diagnosis and Therapy, Sun Yat-Sen University Cancer Center, Guangzhou, 510060 People’s Republic of China; 2 Department of Radiology, Cancer Hospital Chinese Academy of Medical Science, Shenzhen Center, Shenzhen, China

**Keywords:** Rib enhancement, Sclerotic metastasis, Central venous obstruction, Differential diagnosis, Tomography, X-ray computed tomography

## Abstract

**Objective:**

To investigate the CT features of incidental rib enhancement (RE) and to summarize the CT characteristics for distinguishing the RE from sclerotic metastasis (SM) in patients with malignancies.

**Material and methods:**

This retrospective observational study enrolled 79 patients with RE (involved 133 ribs) during October 2014 and December 2021. Another 53 patients with SM (160 SM) in the same period were selected randomly for comparison. The location, enhancement patterns of RE were reviewed. The CT values of RE regions and SM were measured and statistically analyzed.

**Results:**

Most REs (70 patients, 88.6%) were in the 1st to 6th ribs. 50 patients had solitary RE and 29 with multiple REs in a regional distribution. All the REs were closely connected to the intercostal venous plexus (ICVP) ipsilateral to the injection site. No visible abnormalities on unenhanced scans were detected in all REs. One hundred and twenty REs (90.2%) had nodular/patchy enhancement. The CT value of RE regions in the venous phase was lower than that in the arterial phase (589.8 ± 344.2 HU versus 1188.5 ± 325.3 HU, *p* < 0.001). During the venous phase, most REs (125, 94.0%) shrank or disappeared. SM appeared similar on both contrast-enhanced and unenhanced scans in terms of shape and CT values.

**Conclusion:**

The RE demonstrated characteristic CT features. The manifestations of nodular/patchy enhancement in the arterial phase, decreased density and shrinkage or disappearance during the venous phase, and no abnormality on unenhanced scans, as well as a close connection with the ICVP, may help differentiate RE from SM.

**Supplementary Information:**

The online version contains supplementary material available at 10.1007/s00256-024-04609-3.

## Background

Pulmonary or mediastinal malignancies commonly result in central venous obstruction (CVO) due to either a tumor or simple thrombus. According to previous studies [1–4], when contrast agent is injected through the ipsilateral arm to the obstructed central veins, contrast is compelled to flow into the dilated paravertebral collateral veins and intravertebral venous system due to increased venous pressure. Finally, contrast stagnation in the intravertebral venous system induced intravertebral enhancement [1–9], with a reported incidence rate of 11.8% in patients with CVO. In clinical practice, we reviewed patients with CVO, and observed analogous rib enhancement (RE) on enhanced chest CT when contrast reflux into the dilated intercostal venous plexus (ICVP) occurred. Incidental patchy intravertebral enhancement or RE may simulate sclerotic metastasis, which may be misdiagnosed as new bone metastasis and tumor progression, leading to inappropriate clinical treatment. The CT appearance of intravertebral enhancement has been well-studied, whereas there are no prior studies on RE.

Thus, this retrospective observational study aimed to investigate the prevalence and CT features of RE in malignant patients with CVO, and to summarize the CT hallmarks distinguishing RE from sclerotic metastasis.

## Methods

### Patient selection

Our institutional review board approved the study. Written informed consent was obtained from patients who underwent enhanced CT examinations.

To explore the development of RE, the records of patients with pulmonary or mediastinal malignancies and CVO from October 2014 to December 2021 were reviewed. The detailed inclusion criteria were as follows: patients with pulmonary or mediastinal malignancies with CVO, patients who had chest CT scans with unenhanced, arterial, and venous phase, and patients who had RE on enhanced CT. There were 3133 oncology patients with CVO during this period. Those with other collateral veins but not ICVP (*n* = 991) and those without collateral veins (*n* = 1721) were excluded. Among the patients with ICVP filling with contrast (*n* = 421), 342 patients who did not have RE were excluded. Finally, 79 patients who demonstrated RE (RE group: 57.5 ± 12.3 years, 70 men) on chest CTs were included (Figure S1).

Moreover, to analyze the differing CT features between RE and sclerotic metastasis, 53 patients with malignancies with CVO and sclerotic metastasis (SM) (SM group: 54.8 ± 11.1 years, 35 men) in the same period were selected randomly for comparison.

Clinical data (e.g., sex, age, primary tumor types) were obtained from medical records.

### CT image acquisition

Chest CT was performed using a 64-detector spiral CT system (Discovery CT750 HD, GE System) (RE group, *n* = 27; SM group, *n* = 17), a 96-detector spiral CT system (SOMATOM Force CT, Siemens Healthcare) (RE group, *n* = 29; SM group, *n* = 16), or an 80-detector spiral CT system (UIH uCT 780, United Imaging Intelligence, Shanghai, China) (RE group, *n* = 23; SM group, *n* = 20). The acquisition parameters were as follows: 120 kVp, 150–300 mA of automatic adjustment, slice thickness of 5 mm, and pitch of 0.984:1.

For the baseline staging or efficacy evaluation of the malignant tumors, unenhanced and contrast-enhanced CT scans (30–35 s, arterial phase; 55–60 s, venous phase) were routinely obtained for all patients in our center. Enhanced images were obtained after a bolus intravenous injection of 1.5 ml/kg of nonionic contrast agent (Omnipaque 300, GE Healthcare, or Ultravist 370, Bayer Healthcare) through the antecubital vein at a rate of 3 ml/s, which was reduced to 2.5 ml/s or 2.0 ml/s for patients who had received previous radiotherapy or chemotherapy. The axial and multiplanar reformation images were routinely reconstructed at a slice thickness of 2 mm and an interval of 1 mm. The CT images were reconstructed using Standard (soft) kernel and Bone kernel for the GE system, Br44d kernel and Qr50d kernel for the Siemens system, and B_Soft kernel and B_VSHARP kernel for the United Imaging Intelligence system.

The contrast material was injected via the left (RE group, *n* = 37; SM group, *n* = 23) or right (RE group, *n* = 42; SM group, *n* = 30) antecubital veins.

### Types of CVO

All enrolled patients had CVO. Considering that the ICVP drains into the superior vena cava via the azygos venous system as the main pathway [10–13], the extent of the CVO was classified as follows (Figure S2): type 1, patients with obstruction of the superior vena cava above the azygos arch level, with or without obstruction of the brachiocephalic vein (unilateral or bilateral); and type 2, patients with superior vena cava obstruction straddling the azygos arch, with or without azygos arch obstruction, with or without brachiocephalic vein obstruction (unilateral or bilateral).

The communication between the ICVP and other collateral veins (the paravertebral collateral veins, or the anterior or lateral thoracic venous plexus) were recorded.

### Diagnosis of RE and Sclerotic Metastasis

According to the diagnosis of intravertebral enhancement in previous studies [1–4], we diagnosed RE based on the following findings: no abnormality in unenhanced scans, obvious enhancement in the arterial phase, changes in density and morphology in the venous phase, and no abnormality in other imaging examinations (emission CT [ECT], PET-CT, or MRI) during the same period.

Sclerotic metastases typically present as radiodense bone lesions that are round/nodular with relatively well-defined margins on CT in patients with malignant tumors [4, 14, 15]. To compare the CT features of RE to sclerotic metastasis, osteolytic bone metastases with soft tissue masses were excluded. Benign dense bone lesions (bone island, etc.) were also excluded. All cases were diagnosed according to typical findings of CT, ECT, PET-CT or MRI during the same period and follow-up imaging, in the absence of a pathological diagnosis.

### Imaging analysis

Two experienced authors (one with more than 20 years of experience with subspecialty training in musculoskeletal radiology and one with 8 years of experience) reviewed the CT images and decisions were reached by consensus. The images were reviewed with the mediastinal soft tissue window (window width, 250–350 HU; window level, 30–50 HU), and bone window (window width, 950–1500 HU; window level, 250–400 HU).

The following features were reviewed: (1) the enhancement patterns of the ribs (nodular/patchy, linear, and mixed) in arterial phase; the changes of the morphology and boundary of RE and sclerotic metastasis in venous phase; (2) for each patient, the number and level of involving ribs, left/right/bilateral ribs, anterior/posterior ribs, the shape (swelling, normal) and the cortex (interrupted, normal) of the involving ribs; (3) for patients with multiple involving ribs, unilateral/bilateral, adjacent/discrete.

In the RE group, the CT values of the patchy and mixed RE regions in the unenhanced, arterial phase and venous phase scans; and the surrounding normal bone in unenhanced scan were measured. The largest RE region was measured if there were several RE regions. Linear REs were not measured. The measurement of the CT value is as follows (Figure S3): in the arterial phase, the largest slice of patchy RE is selected, the conformal region of interest is drawn along the edge, and each RE region is measured three times. The average of the three measurements is the CT value. The measurement in unenhanced scans and the venous phase will be consistent with the measurement in the arterial phase in terms of the position and range of the region of interest. The CT value of the surrounding normal ribs is measured within 1 cm around the RE region on the unenhanced image, avoiding the outline of cortical bone. Likewise, all CT values of the sclerotic metastases in the SM group were measured (Figure S3).

Further review was performed to determine whether intravertebral enhancement was present, RE was misdiagnosed on the original imaging report or whether the RE or sclerotic metastasis persisted on follow-up imaging.

### Statistical analysis

The age of patients was compared between the RE and SM groups via the unpaired t-test, and categorical variables were analyzed using Pearson's chi-squared test. The one-way ANOVA test was applied to compare the CT values in three phases in both the RE and SM groups. Tukey's multiple comparison test was employed to compare the differences in the CT values in the RE group. Using the unpaired t-test, the following differences in CT values were compared between and within RE and SM groups (Table S1): between the RE region/ sclerotic metastasis and the surrounding normal rib, the surrounding normal rib, the RE region/ sclerotic metastasis in unenhanced CT, and during the arterial and venous phases.

SPSS version 22 (SPSS Inc.) and GraphPad Prism 9.0 (GraphPad Software Inc.) were applied to analyze the data and create graphs. Statistical significance was set at *p* < 0.05.

## Results

### Patient characteristics

As shown in Table 1, 79 patients with RE were included for further analysis. The incidence rate of RE in patients with malignancy and CVO was 2.5% (79/3133 patients) and 18.8% (79/421 patients) in patients with ICVP.

**Table 1:** Demographic data of the RE and SM groups

Variables	RE group	SM group	*p*-value
Cases, n	79 patients, 133 REs	53 patients, 160 SM	
Sex			0.002
Male	70 (88.6%)	35 (66.0%)	
Female	9 (11.4%)	18 (34.0%)	
Age, years (range)	57.5 ± 12.3 (13–78)	54.8 ± 11.1 (35–83)	0.203
Tumor types			NA
Lung cancer	58	38	
Lymphoma	5		
Thymic carcinoma	4	3	
Esophageal cancer	4		
Nasopharyngeal carcinoma	3	6	
Breast cancer		4	
Other*	5	2	
CVO types			0.071
Type 1	25 (31.6%)	25 (47.2%)	
Type 2	54 (68.4%)	28 (52.8%)	
Injected arm			0.697
Left	37 (46.8%)	23 (43.4%)	
Right	42 (53.2%)	30 (56.6%)	

^*^Other tumor types included: RE group, one case each of seminoma, yolk sac tumor, laryngeal carcinoma, maxillofacial squamous cell carcinoma, and malignant mesothelioma in the abdominal cavity; SM group, one case each of neuroendocrine carcinoma in the mediastinum and small cell carcinoma in the mediastinum

Abbreviations: CVO, central venous obstruction; SM, sclerotic metastasis; RE, rib enhancement

In both RE and SM groups, the patients with CVO type 2 out-numbered those patients with type 1 CVO; the difference was not statistically significant, *p* = 0.071 (Table 1).

### Location and number of involved ribs of RE

**Fig.1:**
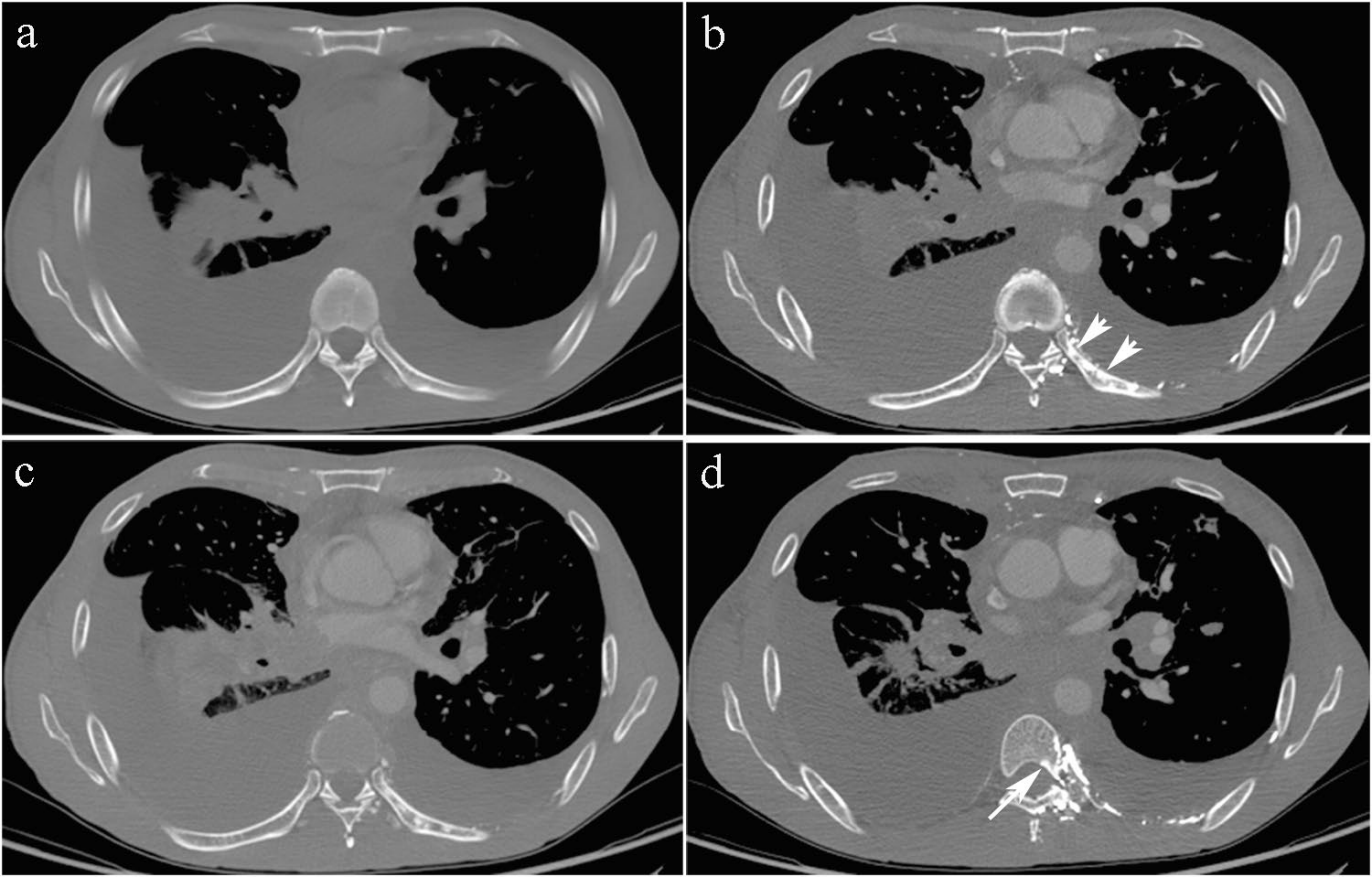
A 48-year-old man was diagnosed with mediastinal lymph node metastases from maxillofacial squamous cell carcinoma. The superior vena cava had an obstruction of type 2. The contrast material was administered via injection into the left arm. The left intercostal venous plexus (ICVP) dilated (**b**) and communicated with the paravertebral collateral veins. Several patchy RE regions were seen in the left 8th posterior rib (**b**, white arrowheads). There was no abnormality on unenhanced scan (**a**). The degree of enhancement decreased and shrank in the venous phase (**c**). Patchy intravertebral enhancement was seen concurrently in the left edge of the 7th thoracic vertebral body (**d**, white arrow). No bone destruction was detected

The RE group involved 133 ribs. As shown in Table 2, 50 patients had solitary rib involvement and 29 had multiple rib involvement (adjacent in 25 patients and discrete in 4). The multiple affected ribs were in a regional distribution for each patient (36 patients had posterior rib involvement, 43 had anterior rib involvement). There were several RE regions in each rib for 13 of the affected ribs (Fig. 1b).

**Table 2:** Comparison of the CT features of RE and Sclerotic Metastasis

Variables	RE group (*n* = 79)	SM group (*n* = 53)	*p*-value
The number of involving ribs per patients, median (range)	1 (1–6)	2 (1–9)	
Solitary	50 patients	17 patients	
Multiple	29 patients	36 patients	
Location			
Left ribs	37 patients (69 RE)	79 SM	
Right ribs	42 patients (64 RE)	81 SM	
Anterior ribs	43 patients (63 RE)	68 SM	
Posterior ribs	36 patients (70 RE)	92 SM	
Patients with multiple ribs involved			
Unilateral / bilateral	29 patients / 0	7 patients / 29 patients	
Adjacent / discrete	25 patients / 4 patients	3 patient / 33 patients	
In a regional distribution	29 patients	0	
Ipsilateral to the injection site			< 0.0001
Yes / No	133 RE / 0	73 SM / 87 SM	
Connected with the ipsilateral ICVP			< 0.0001
Yes / No	133 RE / 0	0 / 160 SM	
Normal unenhanced CT			< 0.0001
Yes / No	133 RE / 0	0 / 160 SM	
Degree of enhancement decreases in the venous phase			< 0.0001
Yes / No	133 RE / 0	0 / 160 SM	
Range changes in the venous phase			< 0.0001
Yes / No	133 RE / 0	0 / 160 SM	
Normal shape of the involving ribs			< 0.0001
Yes / No	133 RE / 0	55 SM / 105 SM	

Abbreviations: CT, Computed tomography; SM, sclerotic metastasis; RE, rib enhancement

Most REs (70 patients, 88.6%) involved the 1st to 6th ribs (Figure S4). The level of affected ribs in cases of RE was not related to the types of CVO, *p* = 0.197 (Table S2).

All the REs were closely connected to the ipsilateral ICVP. Both the RE region and the ICVP were ipsilateral to the injection site. The corresponding ICVP communicated extensively with the anterior/lateral thoracic venous plexus for RE in the anterior ribs or with the paravertebral collateral veins for RE in the posterior ribs. The majority of patients (52/79 patients, 65.8%) with RE concurrently demonstrated intravertebral enhancement of the vertebral body (Fig. 1d), which were ipsilateral to the injection site.

The SM group involved 160 ribs, of which 17 patients had solitary rib involvement and 36 patients had multiple rib involvement. The patients with multiple sclerotic metastases were found to have varied locations: discrete in 33 patients (91.7%) and adjacent in 3 patients, bilateral in 29 patients (80.6%) and unilateral in 7 patients. A localized ICVP was found in 10 patients, which was located level to the 1st to 6th ribs and were ipsilateral to the injection site; however, none of the sclerotic metastases were connected to the ICVP.

### Enhancement patterns of RE

**Fig. 2:**
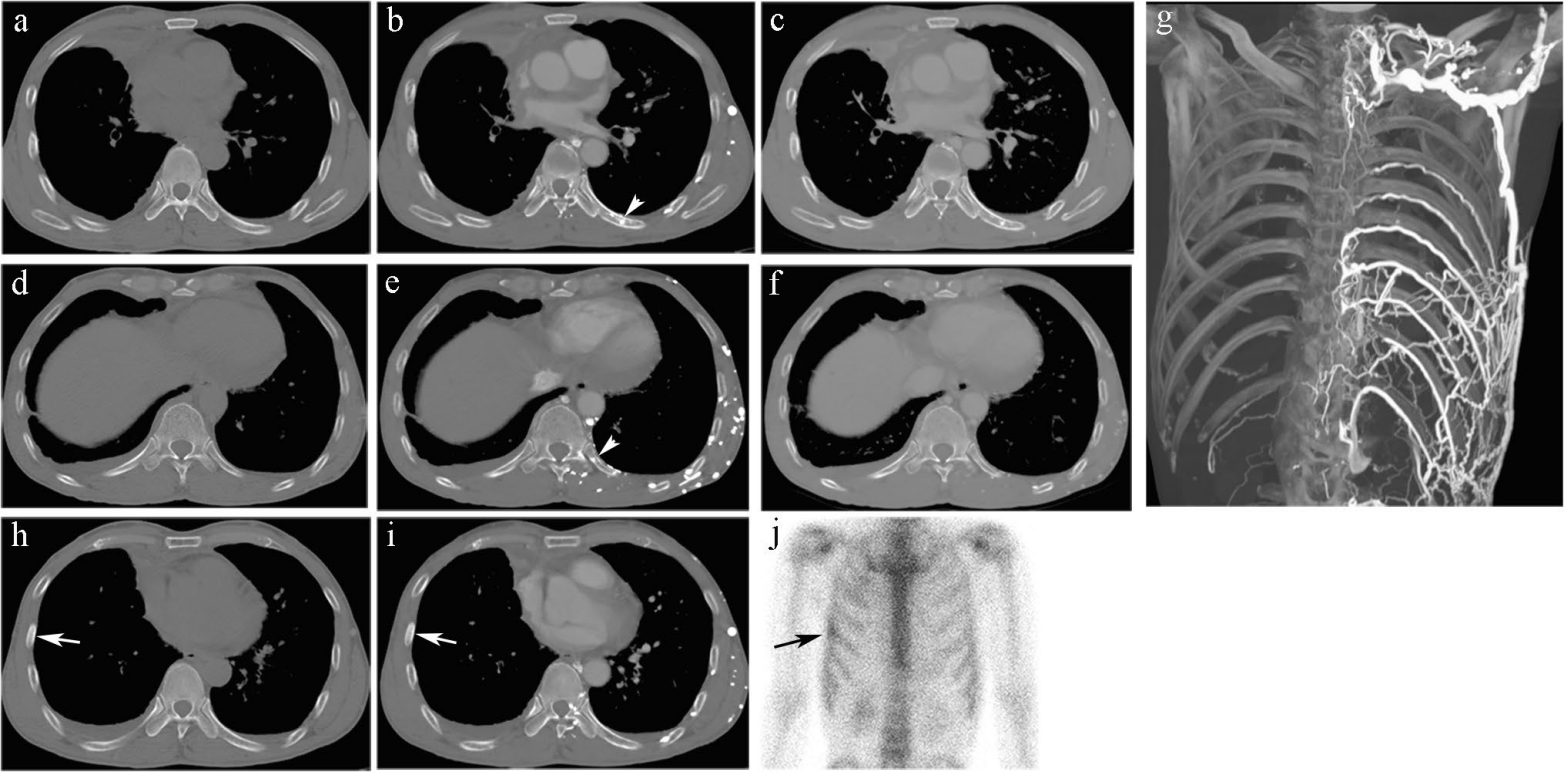
A 53-year-old man with a diagnosis of right central-type lung cancer and right hilar and mediastinal lymph node metastases, complicated by a type 2 CVO. The contrast material was administered via injection into the left arm. The left lower ICVP filled with contrast (**b**) and communicated with the anterior/lateral thoracic venous plexus (**g**, Maximum Density Projection). Patchy /nodular REs were seen in the left eighth (**b**, white arrowhead) and 11th posterior ribs (**e**, white arrowhead). There was no abnormality on unenhanced scan (**a** and **d**) and the degree of enhancement decreased and shrank /disappeared in the venous phase (**c** and **f**). Patchy sclerotic metastasis was detected in the right sixth rib (**h**, unenhanced CT, white arrow; **i**, arterial phase). The sclerotic metastasis showed radioactivity concentration on ECT (**j**) performed within an interval of 2 days, while no visible abnormality was detected in the RE regions

In the RE group during the arterial phase, 120 REs (90.2%) presented as nodular/patchy enhancements (Fig. 1, Fig. 2), 12 were linear, and 1 had a mixed pattern. During the venous phase, the degree of enhancement of the RE region decreased and its shape changed; among them, the range increased in 8 REs and their boundaries became blurred. The other 125 REs (94.0%) shrank or disappeared (Table 3). There was no abnormality in the shape and cortex of the ribs, and no soft tissue mass in the RE region.

**Table 3:** Enhancement patterns of RE in the arterial phase and the changes in the venous phase

Enhancement patterns	The range in venous phase			Total
	Increased	Shrank	Disappeared	
Nodular / patchy	8	85	27	120
Linear		2	10	12
Mixed		1		1

In the SM group, all 160 sclerotic metastases were nodular/patchy. The sclerotic metastases appear similar on both contrast-enhanced and unenhanced CT. Among them, 55 (34.4%) affected ribs showed expansive bone destruction.

### The CT value of RE regions and Sclerotic Metastasis

**Fig.3:**
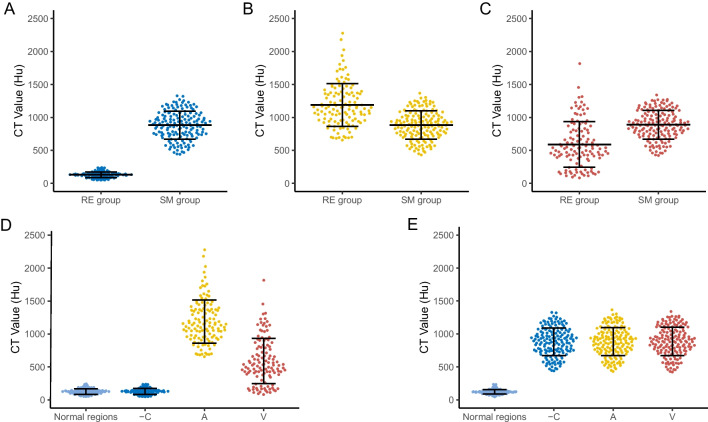
Comparison of the CT value of the RE regions and sclerotic metastasis. (**A**-**C**) Between the RE and SM groups, the CT value of the RE region was higher than that of the sclerotic metastasis during the arterial phase (**B**, *p* < 0.0001) and were lower than that of sclerotic metastasis in unenhanced CT (**A**, *p* < 0.0001) and the venous phase (**C**, *p* < 0.0001). (**D**) In the RE group, the CT value of the RE region in the arterial phase was higher than that in the unenhanced CT (*p* < 0.001) and the venous phase (*p* < 0.001). There was no difference in CT value between the RE region and surrounding normal ribs in the unenhanced CT, *p* = 0.677. (**E**) In the SM group, there was no statistically significant differences in CT values of the three phases, *p* = 0.96. RE, rib enhancement; SM, sclerotic metastasis; − C, unenhanced CT; A, arterial phase; V, venous phase

As shown in Table 4 and Fig. 3d, in the RE group, the CT value of the RE region in the arterial phase was higher than that in the unenhanced scan (1188.5 ± 325.3 HU versus 129.1 ± 43.5 HU, *p* < 0.001), the CT value in the venous phase (589.8 ± 344.2 HU) was lower than that in the arterial phase (*p* < 0.001). The differences in CT values of the three phases were statistically significant (*p* < 0.001). It should be noted that in the RE group, there was no difference in CT value between the RE region and surrounding normal ribs in unenhanced scans (*p* = 0.677).

**Table 4:** Comparison of the CT value of the RE region and Sclerotic Metastasis

	CT value (mean ± SD, HU)			*p*-value
	RE group	SM group		
Unenhanced phase	129.1 ± 43.5	881.8 ± 211.3		< 0.0001
Arterial phase	1188.5 ± 325.3	883.4 ± 214.0		< 0.0001
Venous phase	589.8 ± 344.2	888.3 ± 217.0		< 0.0001
Surrounding normal ribs in unenhanced phase	126.8 ± 40.2	121.8 ± 34.6		= 0.26

The CT values of the patchy and mixed RE region (121 RE) were measured, and 12 linear RE was not measured. All the sclerotic metastases were measured

Abbreviations: CT, Computed tomography; HU, Hounsfield scale; SM, sclerotic metastasis; RE, rib enhancement

As shown in Table 4 and Fig. 3a-c, between the RE and SM groups, the CT value of the RE region was higher than that of sclerotic metastasis in the arterial phase (1188.5 ± 325.3 versus 883.4 ± 214.0,* p* < 0.0001) and lower than that of sclerotic metastasis in the unenhanced scan (129.1 ± 43.5 versus 881.8 ± 211.3,* p* < 0.0001) and during the venous phase (589.8 ± 344.2 versus 888.3 ± 217.0,* p* < 0.0001). However, the difference between the two groups in the CT value of the surrounding normal ribs was not statistically significant (*p* = 0.26).

In the SM group, there was no statistically significant difference in CT values of the three phases (*p* = 0.96; Table 4 and Fig. 3e). The CT value of the sclerotic metastasis was significantly higher than that of the surrounding normal ribs in unenhanced scan (*p* < 0.001).

### Imaging follow-up of the RE group

For further tumor evaluation in the RE group, bone scan (*n* = 10), PET/CT (*n* = 3) or MRI (*n* = 1) were performed within a median interval of 2 day (0–26 days) from CT scanning. No abnormal bony uptake was observed in the RE regions. Thirty-nine patients were followed up with CT imaging for 1.4 − 74.3 months throughout the study period. On follow-up CT, 22 patients were found to have RE in similar regions when the injection site was ipsilateral to the venous obstruction. As mentioned above, no visible abnormalities on unenhanced images were detected in the RE regions in all 79 patients.

In the RE group, 21 patients had bone metastases involving the ribs (Fig. 2h-2j), vertebral body or sternum. In 17 patients (17/79 patients, 21.5%), nodular REs and intravertebral enhancement were misinterpreted as metastases; among them, 7 patients with bone metastases and 10 patients without. The nodular REs and intravertebral enhancement in another 4 patients without bone metastases were suspected to be indeterminate lesions.

In the SM group, 47 patients (88.7%) had multiple detectable sclerotic metastases in other locations, except rib metastases. For further tumor evaluation, bone scan (*n* = 4), PET/CT (*n* = 4) or MRI (*n* = 2) were performed within a median interval of 10.5 days (0–19 days) from CT scanning. The sclerotic metastases demonstrated radioactivity concentration. Among the remaining 43 patients, 39 patients were followed up with CT examinations for 1.0–33.1 months, showing sclerotic bone destruction; 1 patient's bone metastasis was persistent from the previous period, and 3 patients had new bone metastases.

## Discussion

In this study, we first described the CT features of RE in patients with malignancies and CVO and compared the CT features with that of sclerotic metastasis. RE is a transient stagnation of contrast in the ribs and creates pseudopathological enhancement on contrast-enhanced CT. No visible abnormalities on unenhanced CT were detected in the RE regions, which can be used to distinguish RE from sclerotic metastasis.

We found that all the REs were connected to the ICVP, and both the RE region and the ICVP were ipsilateral to the injection site. We postulate that RE occurs through the following mechanism: depending on whether the CVO was type 1 or 2, if there is venous obstruction ipsilateral to the site of injection, an increase in pressure of the distal obstructed veins [3, 4] or a blocked drainage via the azygos vein would result. Subsequently, reflux of the contrast agent into the ICVP would occur, causing RE. Our findings showed that the majority of REs were in ribs 1–6, probably due to injection of contrast agent into the antecubital vein and the higher pressure of the upper ICVP. Due to the extensive communicating collateral branches [10–12], patients with multiple rib enhancements tend to have a regional distribution with more than two drainage veins involved. The mechanism of RE is similar to that of intravertebral enhancement [3, 4]. The dilated ICVP communicates extensively with the paravertebral collateral veins; reflux of contrast could occur in the paravertebral collateral veins as well, resulting in the development of intravertebral enhancement. More than three quarters of patients with RE were found to have simultaneous intravertebral enhancement in this study. According to our results, all REs were connected to the dilated ICVP; however, when there is ICVP, RE does not necessarily occur, and the CVO type cannot predict the location of RE. During follow-up, RE may occur repeatedly or at intervals, depending on whether the embolism persists and whether the injection is administered ipsilateral to the venous occlusion. However, sclerotic metastasis was not associated with the ICVP, injection site, or CVO type.

In theory, RE is a transient stagnation of contrast in the ribs during the arterial phase, similar to that observed in intravertebral enhancement [1–4]. During the venous phase, the degree of enhancement will decrease with the clearance of the contrast agent, and the shape and range will change (most of them will shrink or disappear). For the baseline staging or efficacy evaluation of the malignancies, arterial phase and venous phase were routinely obtained in our center. Therefore, for the patient who performed a venous phase scan, the changes of the density, shape, and range of the RE region during venous phase are also an important clue to differentiate RE from sclerotic metastasis. In summary, RE may be diagnosed when contrast-enhanced CT in patients with CVO reveal the following characteristics: venous obstruction and dense ICVP dilation ipsilateral to the injection site, patchy enhancement during the arterial phase, solitary or multiple with a regional distribution (such as in the anterior or posterior ribs), decrease on the degree of enhancement, change of shape and range (shrinkage or disappearance) in the venous phase, and no abnormality detectable on unenhanced scans.

Radiologists and oncologists are familiar with the imaging signs of sclerotic metastasis, so this study focuses on describing the location and CT features of RE, and summarizes the differential signs between RE and sclerotic metastasis. Incidental patchy RE was easily misdiagnosed as sclerotic metastasis, regardless of whether there were metastases in other locations; this resulted in a misdiagnosis in approximately one fifth of cases in this study. As shown by an earlier investigation [16], patients with non-small-cell lung cancer develop osteosclerotic changes in subtypes with good therapeutic response after chemotherapy, which may reflect the recovery process of osteolytic bone metastasis. RE may also be confused with the osteosclerotic changes. The following features can help to differentiate RE from sclerotic metastasis: 1) The direct signs of RE were nodular/patchy enhancement during the arterial phase, decreased density and shrinkage or disappearance in the venous phase, and no abnormality detectable on unenhanced scans; while the sclerotic metastasis appear similar on both contrast-enhanced and unenhanced CT. It may show expansive bone destruction on CT and increased metabolic activity on ECT or PET-CT. In contrast, there is no abnormality detected on unenhanced CT, ECT, or PET-CT for RE. 2) In addition to the location of involved ribs, the most important indirect signs are that RE is closely connected to the ICVP ipsilateral to the injection site and in our study showed a regional distribution in multiple RE. However, there was no correlation between sclerotic metastasis and the ICVP, most of which were bilateral and disordered. Fully understanding the imaging features of RE would help radiologists and clinicians to distinguish it from sclerotic metastasis, especially those in the clinical practice of oncology, thus reducing excessive staging at baseline and preventing misdiagnosis of tumor progression during follow-up due to the misinterpretation of RE.

Our study had several limitations. First, it was a retrospective study, and the overall sample size was small due to the low incidence of RE. From the above content, the CT value, morphology, and location of RE and sclerotic metastasis demonstrated significant differences, as well as the small number of cases, therefore, the diagnostic accuracy was not evaluated in this study. Second, there could have been selection and verification biases. Although only a small number of patients underwent ECT, PET-CT or MRI examination, most patients had follow-up CT scans; moreover, all patients in the RE group had non-contrast CT scans, and the corresponding regions of the REs were normal. Therefore, verification biases in our study were largely avoided.

## Conclusions

The incidence of RE is low in this study. The RE demonstrated characteristic CT features and accompanying signs. The manifestations of nodular/patchy enhancement in the arterial phase, decreased density and shrinkage or disappearance during the venous phase, and no abnormality on unenhanced scans, as well as the presence of a connection with the ICVP and ipsilateral to the injection site, may help differentiate RE from sclerotic metastasis.

### Supplementary Information

Below is the link to the electronic supplementary material.

Supplementary file1 (DOCX 27 KB)

## Data Availability

The datasets used and/or analyzed during the current study are available from the corresponding author on reasonable request.

## Abbreviations

CVO: Central venous obstruction
ECT: Emission computer tomography
HU: Hounsfield scale
ICVP: Intercostal venous plexus
SM: Sclerotic metastasis
RE/s: Rib enhancement/s

## References

[1] Rasselet B, Larbi A, Viala P (2017). Prevalence and characteristics of intravertebral enhancement on contrast-enhanced CT scans in cancer patients. Eur J Radiol.

[2] Simeone FJ, Bennett DL, Chang CY (2016). Retrospective analysis of intravertebral collateral enhancement in patients with central venous obstruction. Skeletal Radiol.

[3] Kara M, Pradel C, Phan C (2016). CT Features of Vertebral Venous Congestion Simulating Sclerotic Metastases in Nine Patients With Thrombosis of the Superior Vena Cava. AJR Am J Roentgenol.

[4] Kim YK, Sung YM, Hwang KH (2015). Pseudopathologic vertebral body enhancement in the presence of superior vena cava obstruction on computed tomography. Spine J: Official J North Ame Spine Soc.

[5] De Maeyer N, De Wever W, Deroose CM, Vansteenkiste J (2016). A 65-Year-Old Patient with Superior Vena Cava Syndrome and Bone Metastases. J Thorac Oncol.

[6] Berritto D, Abboud S, Kosmas C (2015). Vertebral body enhancement mimicking sclerotic osseous lesions in the setting of bilateral brachiocephalic vein thrombosis. Skeletal Radiol.

[7] Alili C, Larbi A, Thouvenin Y (2013). Transient high density vertebral bone lesions. Skeletal Radiol.

[8] Thomas N, Oliver TB, Sudarshan T (2011). Vanishing bone metastases–a pitfall in the interpretation of contrast enhanced CT in patients with superior vena cava obstruction. Br J Radiol.

[9] Jesinger RA, Huynh B, Gover D (2009). Superior vena cava syndrome resulting in osseous venous congestion simulating sclerotic bone lesions. AJR Am J Roentgenol.

[10] Meier A, Alkadhi H (2019). Venous Collateral Pathways in Superior Thoracic Inlet Obstruction: A Systematic Analysis of Anatomy, Embryology, and Resulting Patterns. AJR Am J Roentgenol.

[11] Marini TJ, Chughtai K, Nuffer Z (2019). Blood finds a way: pictorial review of thoracic collateral vessels. Insights Imaging.

[12] Gosselin MV, Rubin GD (1997). Altered intravascular contrast material flow dynamics: clues for refining thoracic CT diagnosis. AJR Am J Roentgenol.

[13] Kang, O., Bell, D. Venous drainage of the thoracic wall. Reference article, Radiopaedia.org. (accessed on 03 Jul 2022) 10.53347/rID-49548.

[14] Coleman RE, Croucher PI, Padhani AR (2020). Bone metastases Nat Rev Dis Primers.

[15] Yap, K., Knipe, H. Sclerotic bone metastases. Reference article, Radiopaedia.org. (accessed on 21 Jun 2022) 10.53347/rID-10490.

[16] Rong D, Mao Y, Yang Q (2018). Early osteosclerotic changes predict chemotherapy response in non-small-cell lung cancer patients with bone metastases. Eur Radiol.

