# Supplementary material for: Feasible CT features to distinguish incidental rib enhancement from sclerotic metastasis in patients with malignancies
Source: Skeletal Radiol. 2024 Feb 16;53(8):1507–15. doi: 10.1007/s00256-024-04609-3 (PMC11194195; doi:10.1007/s00256-024-04609-3)
Supplement: Supplementary file 1 — Supplementary file1 (DOCX 27 KB) [file 256_2024_4609_MOESM1_ESM.docx]

**Correlation between the level of involving ribs of RE and the types of CVO**

According to the anatomy of the posterior intercostal venous reflux system [1-4], the level of those ribs with enhancement, whether single or multiple, were classed as: rib 1 on the left, ribs 2–4 on the left, and rib 1 on the right (the affected posterior intercostal veins did not drain into the azygos vein), and ribs 5–12 on the left and ribs 2–12 on the right (the affected posterior intercostal veins did drain into the azygos vein).

The correlation between the level of affected ribs with enhancement and the types of CVO were analyzed using Pearson’s chi-squared test.

Results showed that the level of the affected ribs with enhancement was not related with the types of CVO, p=0.197 (Table S2). In patients with RE involving the posterior intercostal veins draining into the azygos vein, 16/58 patients (27.6%) had CVO type 1. While in patients with CVO type 2, 12/54 patients (22.2%) had RE affecting only ribs 1 and 2 on the left, and rib 1 on the right.

As shown in Figure S4, in patients with multiple REs (n=29), 8 patients had REs located in left 2–8 ribs, the affected posterior intercostal veins drained into both the left superior intercostal vein and the accessory hemiazygos veins; 1 patient had involvement of the 8th and 11th ribs of which the affected posterior intercostal veins drained into the accessory hemiazygos and the hemiazygos veins; 5 patients had REs located in right 1-5 ribs, the affected posterior intercostal veins drained into both the right supreme intercostal vein and the azygos vein. In the remaining 15 patients, the drainage of the affected posterior intercostal veins was in an anatomic distribution, i.e., the left/right superior intercostal vein, the hemiazygos vein, or the azygos vein.

**References**

1. Meier A, Alkadhi H (2019) Venous Collateral Pathways in Superior Thoracic Inlet Obstruction: A Systematic Analysis of Anatomy, Embryology, and Resulting Patterns. AJR Am J Roentgenol 213:200-210.

2. Marini TJ, Chughtai K, Nuffer Z, Hobbs SK, Kaproth-Joslin K (2019) Blood finds a way: pictorial review of thoracic collateral vessels. Insights into imaging 10:63.

3. Gosselin MV, Rubin GD (1997) Altered intravascular contrast material flow dynamics: clues for refining thoracic CT diagnosis. AJR Am J Roentgenol 169:1597-1603.

4. Kang, O., Bell, D. Venous drainage of the thoracic wall. Reference article, Radiopaedia.org. (accessed on 03 Jul 2022) <https://doi.org/10.53347/rID-49548>.

**Table S1** Statistical analysis between and within RE and OM groups

| **Comparing variables** | **Statistical analysis** |
| --- | --- |
| In the RE group: CT values in three phases | One-way ANOVA test |
| In the OM group: CT values in three phases |  |
| Within the RE group:  CT values in the arterial phase vs. unenhanced CT,  CT values in the arterial phase vs. venous phase | Tukey's multiple comparison test |
| RE vs OM region in unenhanced CT | Unpaired t test |
| RE vs OM region in the arterial phase |  |
| RE vs OM region in the venous phase |  |
| RE region in unenhanced CT vs surrounding normal rib |  |
| OM region in unenhanced CT vs surrounding normal rib |  |
| Surrounding normal rib in unenhanced CT between RE and OM group |  |

Abbreviations: CT, Computed tomography; OM, osteosclerotic metastasis; RE, rib enhancement.

**Table S2** Correlation between the level of affected ribs of RE and the types of CVO

| **The level of affected ribs with RE** | **The CVO types** | | ***p*-value** |
| --- | --- | --- | --- |
|  | Type 1 | Type 2 |  |
| Left 1, left 2–4, and right 1 rib | 9 | 12 | 0.197 |
| left 5–12, and right 2–12 ribs | 16 | 42 |  |

Abbreviations: CVO, central venous obstruction; RE, rib enhancement.
